# *Geobacter metallireducens*—evaluation of its sustainability in nitrate-reducing Fe(II) oxidation under autotrophic conditions

**DOI:** 10.1093/femsec/fiag098

**Published:** 2026-08-29

**Authors:** Stefanie Becker, Lukas Ostler, Andreas Kappler

**Affiliations:** Geomicrobiology, Department of Geosciences, University of Tübingen, Schnarrenbergstrasse 94‒96, D-72076 Tübingen, Germany; Geomicrobiology, Department of Geosciences, University of Tübingen, Schnarrenbergstrasse 94‒96, D-72076 Tübingen, Germany; Geomicrobiology, Department of Geosciences, University of Tübingen, Schnarrenbergstrasse 94‒96, D-72076 Tübingen, Germany

**Keywords:** nitrogen cycle, iron cycle, chemolithoautotroph, microbial Fe(II) oxidation, non-autotrophic nitrate-reducing Fe(II) oxidation, *Geobacter metallireducens*

## Abstract

Bacterial nitrate (NO_3_^−^) -reducing Fe(II) oxidation is often regarded as an autotrophic process, although its long-term sustainability is rarely demonstrated. Here, we assessed whether *Geobacter metallireducens* (ATCC 53774) can sustain chemolithoautotrophic nitrate-reducing Fe(II) oxidation. Cultures were incubated with Fe(II) and NO_3_^−^ as electron donor and acceptor, respectively, and CO_2_ as the sole carbon source. Negative controls contained either (1) electron acceptor, (2) donor or (3) no substrates. We found that first-generation cultures transferred from a washed NO_3_^−^/acetate-grown preculture oxidized 21.0±1.3% (2.09±0.14 mM) of 10 mM Fe(II) and reduced 5.2±3.9% (0.20±0.15 mM) of 4 mM NO_3_^−^. Nitrite remained below the detection limit and ammonium increase was not observed while total N_2_O accumulated up to 31.34±1.90 µM. However, following transfers under organic-free conditions did not sustain Fe(II) oxidation. Further, we did not observe a decrease in nitrate, although we found low N_2_O formation of total 9.10±0.42 µM and 14.10±3.90 µM in later generations. These results demonstrate that *G. metallireducens* does not sustain autotrophic nitrate-reducing Fe(II) oxidation, although it may contribute to coupled Fe(II) oxidation and nitrate reduction in organic environments.

## Teaser

Transfer experiments under chemolithoautotrophic conditions demonstrate that *Geobacter metallireducens* cannot sustain nitrate-reducing Fe(II) oxidation, suggesting that it likely contributes to Fe(II) oxidation only in organic environments.

## Introduction

Bacterial nitrate-reducing Fe(II) oxidation is an important environmental process linking the nitrogen and iron geochemical cycles (Kappler et al. 2021). Nitrate reduction produces climate-relevant gases such as N₂O and NO as intermediates (Qi et al. 2025). N_2_O is a potent greenhouse gas due to its long atmospheric residence time (∼109 years) and strong infrared absorption capability (Forster et al. 2021). Furthermore, photolysis of N_2_O in the stratosphere yields active nitrogen oxide radicals (NO_x_), which drive ozone depletion (Ravishankara et al. 2009). Fe(II) oxidation in soils and sediments influences their iron speciation and mineralogy, thereby impacting ecosystem functioning by regulating nutrient availability and heavy metal solubility (Chauhan et al. 2024, Drabesch et al. 2024, Grimm et al. 2024).

Due to the high nitrate load from agriculture and locally high iron occurrence in the environment (Rivett et al. 2008), denitrifying Fe(II)-oxidizers have become a research focus in environmental science and waste water treatment, in particular as a potential eco-friendly strategy for bioremediation of nitrate-polluted groundwater (Zhang et al. 2016, 2018, Jokai et al. 2021). Fe(II) oxidation in the presence of nitrate and denitrifying bacteria may follow two distinct mechanisms. The first mechanism results from bacterial nitrate reduction, which produces reactive nitrogen species that spontaneously oxidize Fe(II) through chemodenitrification (Sørensen and Thorling 1991, Tai and Dempsey 2009, Jamieson et al. 2018). The second mechanism is enzymatic, with denitrifying bacteria directly coupling Fe(II) oxidation to their metabolism (Bryce et al. 2018, Becker et al. 2021). As an umbrella term, bacteria causing one or both mechanisms are often referred to in the literature as nitrate-dependent Fe(II)-oxidizing bacteria (NDFeOx). While this terminology reflects the dependence of Fe(II) oxidation on the presence of nitrate, it may be misleading because it could also be interpreted as a dependence of the bacteria on nitrate as a nitrogen source for assimilation. Therefore, we avoid this terminology. Bacteria carrying out enzymatic Fe(II) oxidation we consistently referred to as nitrate-reducing Fe(II)-oxidizers (NRFeOx), since Fe(II) oxidation is directly linked to nitrate reduction, for example through an electron transfer chain (Becker et al. 2021, 2025, Becker and Kappler 2026).

**Figure 1:**
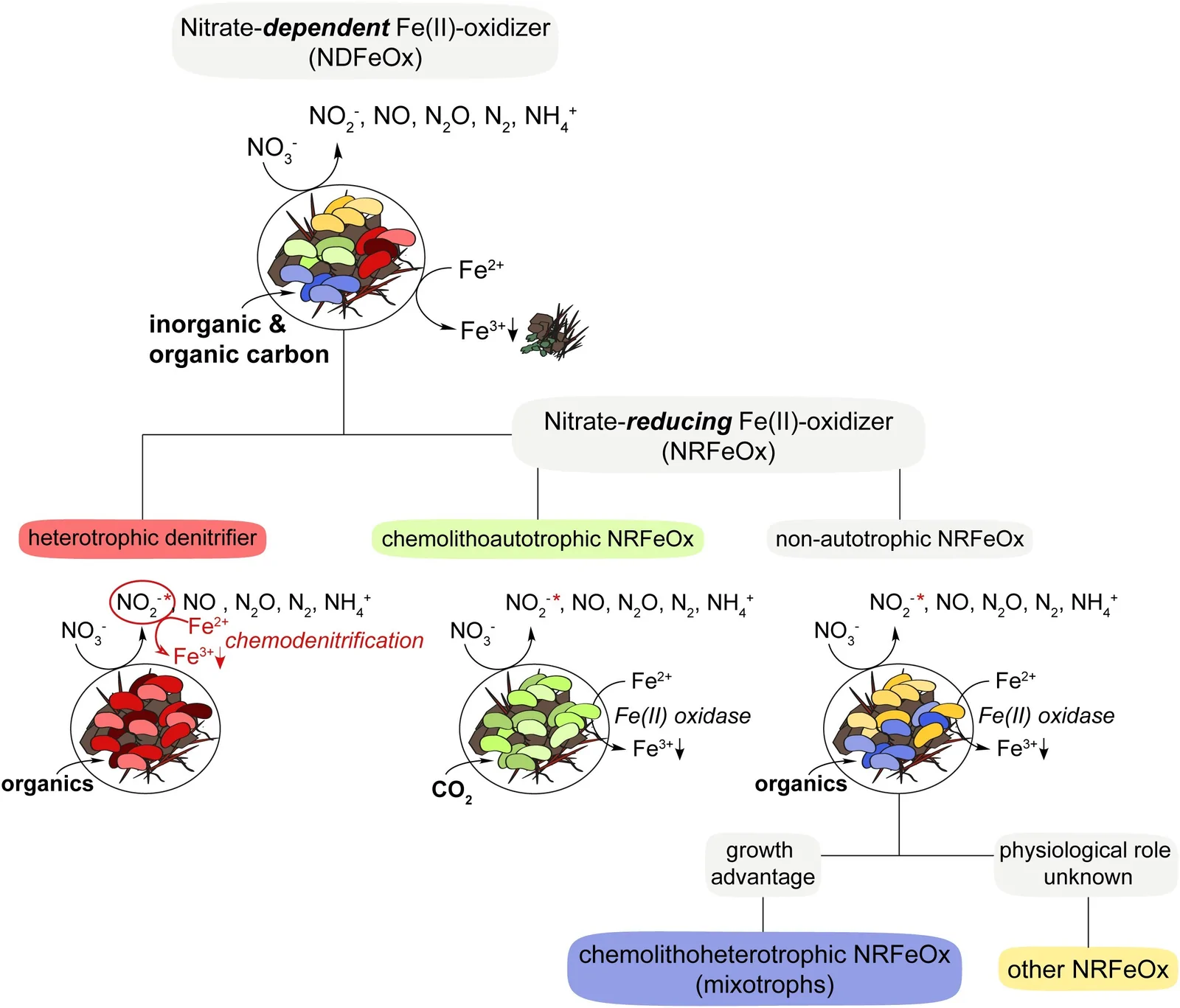
Terminology of nitrate-reducing bacteria causing Fe(II) oxidation. The term nitrate-dependent Fe(II) oxidizer is commonly used to describe these micro-organisms. However, the word *dependent* may be misleading, as it could imply that the bacteria rely on nitrate as a nutrient. In reality, the term refers to the requirement of nitrate for the Fe(II) oxidation process to occur. For this reason, we discourage the use of this terminology. Further, we use nitrate-reducing Fe(II)-oxidizers to discriminate the bacteria that directly oxidize Fe(II) by an enzymatic pathway (chemolithoautotrophic and non-autotrophic NRFeOx) to heterotrophic denitrifies that cause Fe(II) oxidation by chemodenitrification (abiotic process). *Intermediate that may induce chemodenitrification when released by cells.

Overall, denitrifying bacteria associated with Fe(II) oxidation can be categorized into two groups: heterotrophic denitrifiers that cause Fe(II) oxidation solely through chemodenitrification (Fig. 1, red bacteria) and NRFeOx. The latter can be further subdivided into chemolithoautotrophic NRFeOx (Fig. 1, green bacteria) and non-autotrophic NRFeOx. The latter group encompasses (i) chemolithoheterotrophs (often referred to as mixotrophs), whose carbon demand cannot be met by inorganic carbon alone and which depend on organic carbon while benefiting from additional Fe(II) oxidation (Fig. 1, blue bacteria), and (ii) organisms for which the physiological advantage of Fe(II) oxidation remains unclear (Fig. 1, yellow bacteria).

Bacterial strains that exhibit nitrate-reducing Fe(II) oxidation in the absence of organic carbon are often classified as autotrophs [reviewed by Bryce et al. (2018)]. However, most of these strains have not been studied in sufficient detail to verify the sustainability of this metabolism, which is a necessary criterion for such classification (Bryce et al. 2018). One such understudied representative is *Geobacter metallireducens* strain ATCC 53774 (*G. metallireducens*), a Gram-negative Deltaproteobacterium and metabolically versatile anaerobe that plays an important role in iron and nitrogen cycling in anoxic environments. *G. metallireducens* is capable of utilizing a wide range of electron donors including sugars, fatty acids, and aromatic compounds as well as acceptors, including nitrate, Fe(III), uranium, and other heavy metals (Lovley et al. 1987, 1993). A characteristic feature of this organism is its ability to transfer electrons to poorly soluble substrates, enabling respiration with metal oxide minerals (Childers et al. 2002). To facilitate this process, *G. metallireducens* produces conductive pili that promote electron exchange with poorly soluble materials such as Fe(III) (oxyhydr)oxides when soluble electron acceptors are unavailable.

In addition to reducing Fe(III), *G. metallireducens* has been shown to oxidize Fe(II) in the presence of nitrate as an electron acceptor (Weber et al. 2006b, Finneran et al. 2002). In the study of Weber et al., *G. metallireducens* was incubated in acetate/NO_3_^−^/Fe(III)-containing medium leading to the formation of Fe(II). After acetate depletion, nitrate was spiked as a fresh electron acceptor, resulting in rapid Fe(II) oxidation, denitrification, and accumulation of NH_4_⁺ at molar ratios. This approach does not reflect a strict autotrophic condition. Although acetate was depleted from the medium, it had previously been assimilated by the cells and was likely stored intracellularly, for example as fatty acids. In addition, the medium may contain other organic compounds, such as exometabolites released during bacterial metabolism. Consequently, experiments transitioning from organic-rich to autotrophic conditions are not ideal for assessing the physiological capacity for autotrophy. On the other hand, such transitions are often necessary in experimental designs to allow cells to adapt to the new environmental conditions using stored organic carbon.

Here, we conducted an experiment involving multiple transfers of a *G. metallireducens* culture. The first transfer followed growth on acetate/NO_3_^−^-containing medium and therefore contained cells with recently assimilated organic carbon, whereas two subsequent transfers were performed under strictly autotrophic conditions. This approach allowed us to observe Fe(II) oxidation behavior under progressively more autotrophic conditions. In doing so, we assessed the sustainability of nitrate-reducing Fe(II) oxidation in *G. metallireducens* and evaluated whether this organism has the potential to function as a truly autotrophic nitrate-reducing Fe(II)-oxidizer.

## Material and methods

### Experimental setup

**Figure 2:**
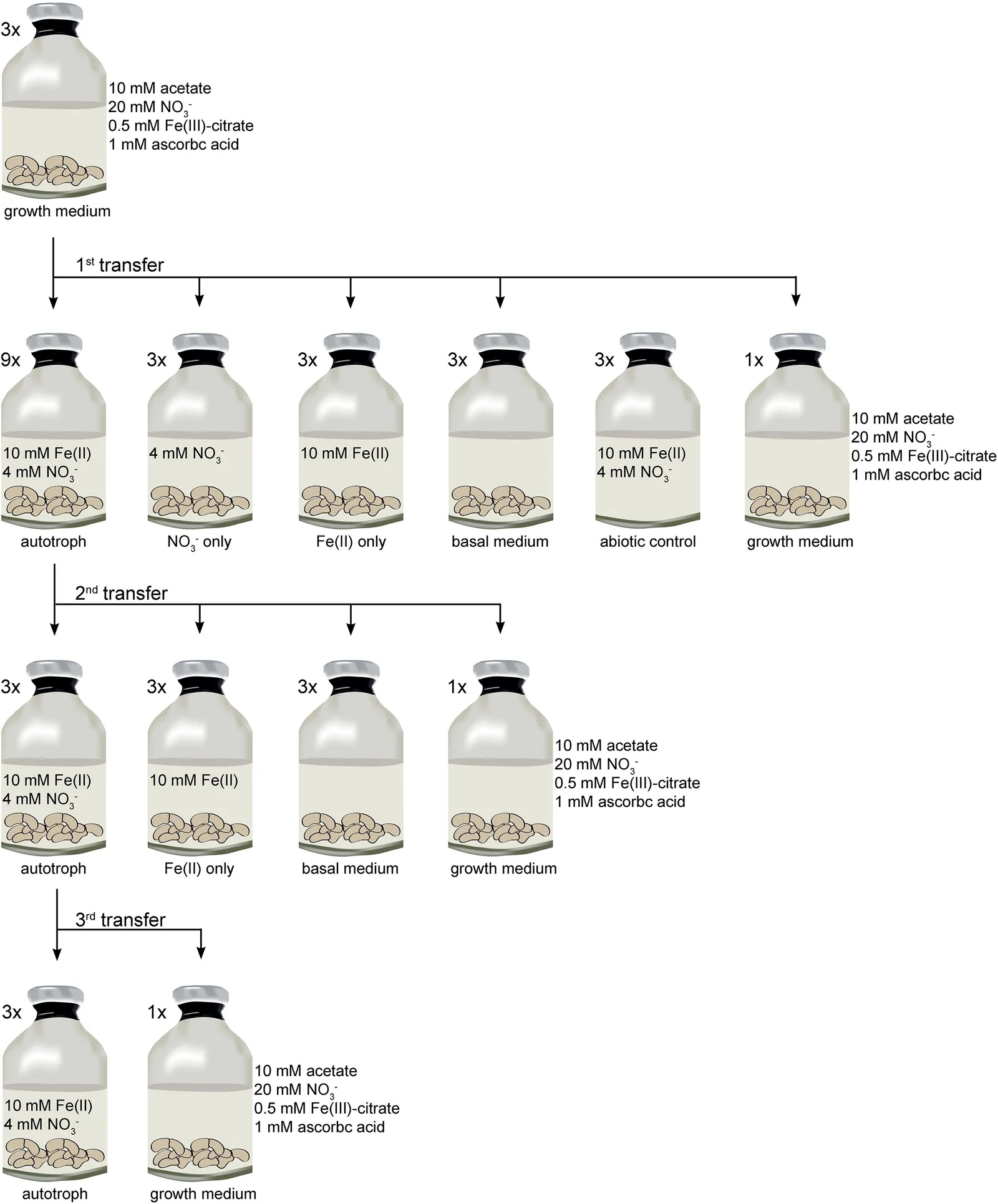
Overview of experimental setups to evaluate the sustainability of autotrophic nitrate-reducing Fe(II) oxidation by *G. metallireducens*. The heterotrophically grown precultures, harvested at the exponential phase, were washed and transferred (first transfer) to Fe(II)-oxidizing denitrifying conditions (autotroph), three negative controls (containing Fe(II), NO_3_⁻or neither of both) and a heterotrophic growth control (growth medium containing acetate and NO_3_⁻) resulting in ∼2.2 × 10^6^ cells ml^−1^ starting concentration. For the second and third transfer autotrophic cultures were harvested, washed, and transferred to fresh medium.

All cultivations were conducted under a 90% N_2_/10% CO_2_ atmosphere at 30°C. The inoculum for the first transfer was 10% (vol/vol) of an NO culture harvested in late exponential phase (∼2.2×10^6^ cells ml^−1^ final concentration). The cells were washed twice with freshwater basal medium lacking Fe(II) and NO_3_^−^, by centrifugation (10 min, 6200 × g and 20°C), then inoculated on freshwater basal medium containing 10 mM Fe(II) (electron donor) and 4 mM NO_3_^−^ (electron acceptor), as well as controls lacking either Fe(II) or NO_3_^−^, or lacking both (first transfer, Fig. 2). After 9 days of incubation, nine cultures containing both Fe(II) and NO_3_^−^ were harvested, washed (10 min, 6200 × g and 20°C) and used as inoculum for fresh medium containing both Fe(II) and NO_3_^−^, only Fe(II) and lacking both substrates (second transfer, Fig. 2). Again, after 9 days of incubation, three cultures of the medium containing both Fe(II) and NO_3_^−^ were harvested, washed (10 min, 6200 × g and 20°C) and used as inoculum for fresh medium containing both Fe(II) and NO_3_^−^ (third transfer, Fig. 2).

Each transfer was accompanied by a positive control using the acetate-/NO_3_^−^-containing growth medium to access the viability of the culture under ideal growth conditions. The growth medium was amended with 0.5 mM Fe(III)-citrate to supply additional iron required for sustained growth during nitrate respiration (Senko and Stolz 2001). In addition, 1 mM ascorbic acid was included to limit the accumulation of NO_2_⁻, which can inhibit growth under nitrate-reducing conditions (Weber et al. 2006b). We monitored inoculated cultures (triplicates) alongside with abiotic controls for NO_3_⁻, NO_2_⁻, N_2_O, NH_4_^+^, Fe(II), and Fe(III) concentrations, cell number (for cultures without iron) and viability.

**Source of micro-organism**. Experiments were conducted with *G. metallireducens* strain ATCC 53774, originally isolated by Derek R. Lovley from alluvial sediments of the Potomac River (Lovley Derek et al. 1987, Lovley et al. 1993).

**Media preparation**. 30 mM bicarbonate buffered growth medium was used for strain maintenance, pre-culture and positive growth control. The medium was made after Weber et al. (2006a) and contained 10 mM acetate, 20 mM NO_3_^−^, 0.5 mM Fe(III)-citrate and 1 mM ascorbic acid as growth supporting substrates.

Fe(II) oxidation, nitrate reduction were evaluated using 30 mM bicarbonate buffered freshwater basal medium (pH 7.24) as described by Bruce et al. (1999) (without NaClO_3_). The trace element solution was prepared after Balch et al. and contained the organic molecule nitrilotriacetic acid (0.078 mM, NTA) functioning as chelator to dissolve components (Balch et al. 1979). Anoxic media preparation followed the instructions of Widdel and Bak (1992). The media were distributed to Schott-bottles using a Widdel-flask. For the experiment, 10 mM FeSO_4_ and 4 mM NaNO_3_ were added to the freshwater basal medium using syringes, according to the different setups as shown in Fig. 1. Fifty millilitres of finalized medium were then distributed into sterile 100 ml serum bottles filled with 90% N_2_/10% CO_2_, using syringes. The initial overpressure of the serum bottles has been adjusted to atmospheric pressure using a gas trap, prior to inoculation with bacteria.

**Sampling procedure**. Samples were withdrawn anoxically through the rubber stopper using a syringe while bottles were gently mixed by hand inside an anoxic glovebox. Subsamples were used for cell enumeration and quantification of Fe_tot_ and Fe(II)_tot_ (including dissolved and solid-phase fractions). The remaining volume was centrifuged at 12 408 × g for 5 min at 25°C, and the supernatant was used for determination of nitrogen species and aqueous Fe(II) [Fe(II)_aq_].

Iron samples were diluted 21-fold in SA: HCl (40 mM amidosulfonic acid, 1 M HCl) (Granger and Sigman 2009, Klueglein and Kappler 2013). Nitrate samples were diluted 21-fold in distilled water, whereas undiluted samples were used for NO₂⁻ quantification. All samples were stored anoxically at 4°C until analysis. Removed liquid and gas volumes were replaced with 90% N₂/10% CO₂.

### Analytical methods

**N₂O** concentrations were determined as described by Grimm et al. (2024). Briefly, 1 ml of headspace gas was transferred into helium-flushed headspace vials, resulting in a 1 : 12 dilution. N₂O was quantified using the front pulsed discharge detector of a TRACE 1310 gas chromatograph (Thermo Fisher Scientific, USA). Calibration standards ranged from 0.05 to 380 ppm, and dissolved concentrations were calculated using Henry’s law constant.

**Fe_tot_, Fe(II)_tot_, and Fe(II)_aq_** were quantified using a colorimetric ferrozine-based microplate assay following the procedure described by Becker et al. using 96-well microplates and a Multiskan GO microplate spectrophotometer (Thermo Fisher Scientific, Inc.) (Stookey 1970, Schaedler et al. 2018, Becker et al. 2025). Relative F e(II)_tot_ (%) was calculated as Fe(II)_tot_/Fe_tot_ × 100. Absolute Fe(II)_tot_ concentrations (mM) were calculated as Fe(II)_tot_/Fe_tot_ × 10 mM, where 10 mM represents the initial Fe(II) concentration.

**NO_3_^−^, NO_2_^−^ and NH_4_^+^** were quantified colorimetrically using a continuous-flow analyzer (AA3, SEAL Analytical, Norderstedt, Germany) according to Becker et al. (2025) (Skeggs 1957, Anderson 1979, Becker et al. 2025).

**Cell numbers** were determined by flow cytometry using an Attune NxT instrument equipped with an autosampler (Thermo Fisher Scientific, Inc.) as described in Becker et al. (2025). Culture samples (100 µl) were diluted with 900 µl 0.9% NaCl followed by successive 10-fold dilutions up to 10³. The fluorescence day was BacLight™ Green Bacterial Stain. Note that we could not optimize the method for bacteria incubated with 10 mM Fe(II).

**Cell viability** was assessed using the LIVE/DEAD™ BacLight™ Bacterial Viability Kit according to the manufacturer’s instructions and examined by fluorescence microscopy using a Leica CTR5500 microscope (Leica Microsystems). Images were processed with Leica Application Suite X.

**Statistics**. Standard deviations were calculated from replicate measurements using the sample standard deviation function in Microsoft Excel (STDEV.S).

## Results and discussion

### Fe(II) oxidation depends on NO_3_⁻ reduction

To evaluate nitrate-reducing Fe(II) oxidation under autotrophic conditions we used five different media: Fe(II)-/NO_3_⁻-containing medium (autotrophic NRFeOx conditions), Fe(II) only medium (control for NO_3_⁻-independent Fe(II) oxidation), NO_3_⁻ only medium (control for Fe(II)-independent NO_3_⁻ reduction), basal medium without electron donor or acceptor (control for growth supported by trace organic carbon), and acetate/NO_3_⁻-containing medium (control for viability of culture). For all setups, heterotrophic denitrifying cultures of *G. metallireducens* were harvested during exponential growth, washed, and transferred into the respective media (first transfer, Fig. 2).

**Figure 3:**
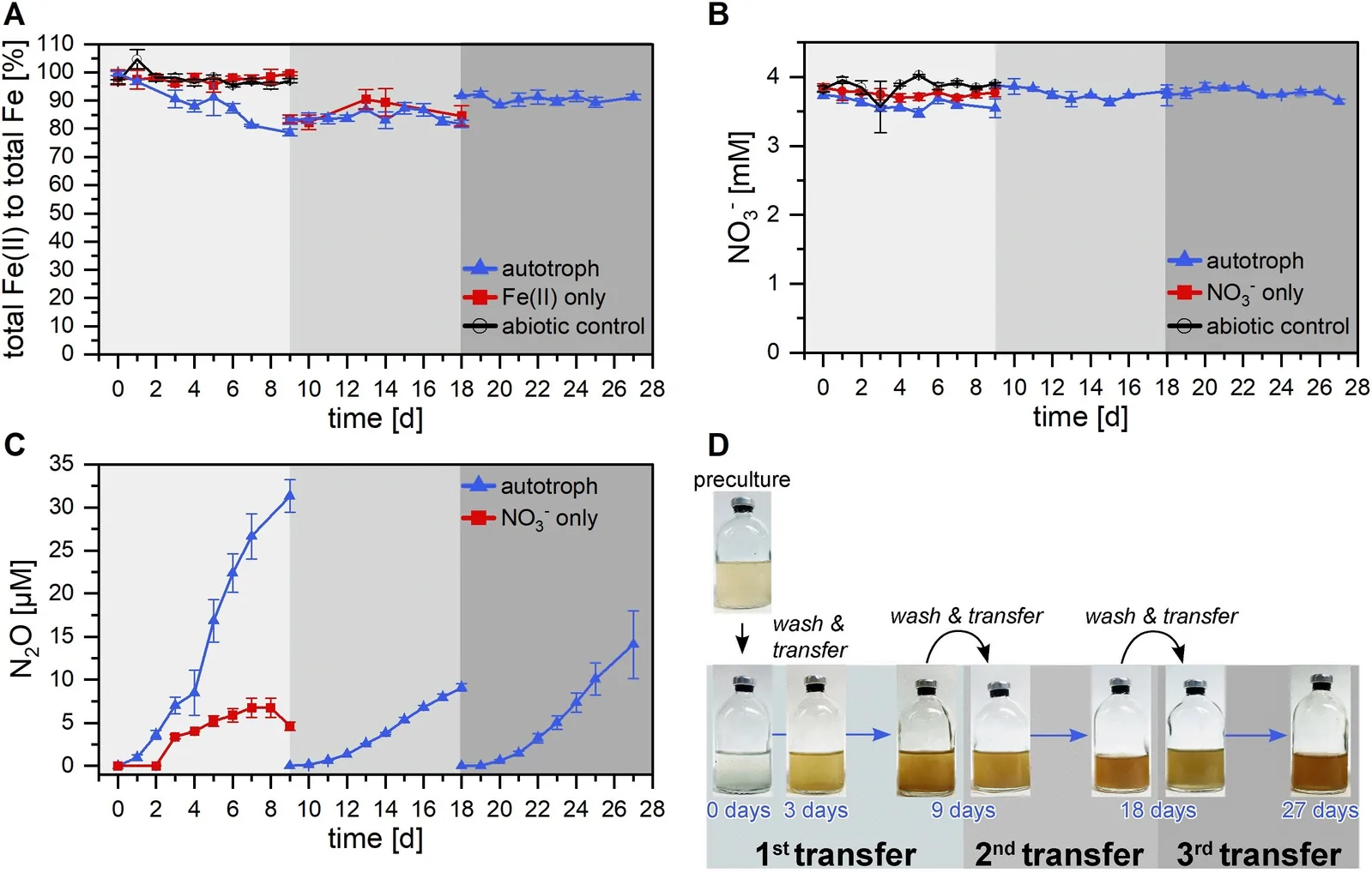
Evaluation of autotrophic NO_3_⁻-reducing Fe(II) oxidation by *G. metallireducens* after transferring heterotrophically grown (medium containing 10 mM acetate and 20 mM NO_3_⁻) and washed cells into autotrophic medium containing 10 mM Fe(II) and 4 mM NO_3_⁻. Light gray background: first transfer from heterotrophically grown preculture. Middle gray background: second transfer from a nitrate-reducing, Fe(II)-oxidizing culture incubated on autotrophic conditions for 9 days. Dark gray background: third transfer from a nitrate-reducing, Fe(II)-oxidizing culture incubated on autotrophic conditions for 18 days. (A) Fe(II) oxidation. (B) Nitrate reduction. (C) N_2_O accumulation. N_2_O measurement of the abiotic control were under the detection limit (data not shown). (D) Schematic of cultivation procedure showing representative bottles of the autotropic condition. Error bars indicate ±1 standard deviation calculated from triplicate measurements. NO_2_⁻ concentrations were below the detection limit (data not shown). NH_4_^+^ data in Fig. S1.

Cultures incubated under autotrophic NRFeOx conditions showed NO_3_⁻-reducing Fe(II) oxidation (Fig. 3A and B) within 9 days after the first transfer. Fe(II) oxidation accounted to 21.0±1.3% (2.09±0.14 mM) of 10 mM total Fe(II), which results in 9.5 × 10^−7^ mmol cell^−1^ (Fig. 3A). NO_3_⁻ reduction was 5.2±3.9% (0.20±0.15 mM) of 4 mM NO_3_⁻ (Fig. 3B). NO_2_⁻ was under the detection limit for all tested conditions. N_2_O accumulated to total 31.34±1.90 µM, corresponding to 32% of the 0.20 mM reduced NO_3_⁻ (Fig. 3C). However, the uncertainty in NO_3_⁻ reduction (±0.15 mM, 74.6%) propagates into a large uncertainty in this percentage, limiting the confidence of the estimate. The ratio of Fe(II)_oxidized_ to NO_3_⁻_reduced_ (Fe_ox_: NO_3_⁻_red_) is 10.6**±**8.0.

Furthermore, we showed that Fe(II) oxidation required the simultaneous presence of NO_3_^⁻^, as no Fe(II) oxidation was observed in the Fe(II) only control, nor was a decrease in NO_3_^⁻^ observed in the NO_3_^⁻^ only control (Fig. 3A and B). However, we did measure accumulation of total N_2_O up to 4.63±0.49 µM (14.77% of N_2_O formed by the first transfer autotrophic condition). This may reflect minor denitrification activity that was not detectable by NO_3_⁻ measurements alone, as changes of this magnitude fall within the analytical uncertainty. The denitrification activity observed in the NO_3_^⁻^ only control was not associated with an increase in cell numbers (Fig. S2A) and therefore was likely the result of cell maintenance (using trace organic carbon in the medium or stored organic carbon in the cells as electron donor) but not proliferation.

Biological denitrification to molecular nitrogen proceeds in four steps (Equations 1‒4) with NO_2_^−^, NO, and N_2_O as intermediates. Although NO_2_^−^ can also react abiotically with Fe(II), this reaction is relatively slow at neutral pH and in bicarbonate-buffered medium, and biological production of NO_2_⁻ would be expected to result in detectable accumulation (Tai and Dempsey 2009, Becker et al. 2025). Because N_2_O formation was higher in the Fe(II)-amended conditions, we consider N₂O to be a product of nitrate-reducing Fe(II) oxidation in *G. metallireducens*.


(1) \begin{eqnarray*}
2{\mathrm{Fe}}\left( {{\mathrm{II}}} \right) + {\mathrm{N}}{{\mathrm{O}}_3}^ - + \ 2{{\mathrm{H}}^ + } \to 2{\mathrm{Fe}}\left( {{\mathrm{III}}} \right) + {\mathrm{N}}{{\mathrm{O}}_2}^ - + {{\mathrm{H}}_2}{\mathrm{O}}
\end{eqnarray*}

(2) \begin{eqnarray*}
{\mathrm{Fe}}\left( {{\mathrm{II}}} \right) + {\mathrm{N}}{{\mathrm{O}}_2}^ - + 2{{\mathrm{H}}^ + } \to {\mathrm{Fe}}\left( {{\mathrm{III}}} \right) + {\mathrm{NO}} + {{\mathrm{H}}_2}{\mathrm{O}}
\end{eqnarray*}

(3) \begin{eqnarray*}
2{\mathrm{Fe}}\left( {{\mathrm{II}}} \right) + 2{\mathrm{NO}} + 2{{\mathrm{H}}^ + } \to 2{\mathrm{Fe}}\left( {{\mathrm{III}}} \right) + {{\mathrm{N}}_2}{\mathrm{O}} + {{\mathrm{H}}_2}{\mathrm{O}}
\end{eqnarray*}

(4) \begin{eqnarray*}
2{\mathrm{Fe}}\left( {{\mathrm{II}}} \right) + {{\mathrm{N}}_2}{\mathrm{O}} + 2{{\mathrm{H}}^ + } \to 2{\mathrm{Fe}}\left( {{\mathrm{III}}} \right) + {{\mathrm{N}}_2} + {{\mathrm{H}}_2}{\mathrm{O}}.
\end{eqnarray*}

Complete denitrification to molecular nitrogen (N_2_) requires 10 electrons derived from Fe(II) (Equation 5), resulting in a theoretical Fe(II) oxidation to NO₃⁻-reduction ratio of 5.


(5) \begin{eqnarray*}
10{\mathrm{Fe}}\left( {{\mathrm{II}}} \right) + 2{\mathrm{N}}{{\mathrm{O}}_3}^ - + 12{{\mathrm{H}}^ + } \to 10{\mathrm{Fe}}\left( {{\mathrm{III}}} \right) + {{\mathrm{N}}_2} + 6{{\mathrm{H}}_2}{\mathrm{O}}.
\end{eqnarray*}

Weber et al. 2006b et al. ( observed an increase in ammonium (NH_4_^+^) concentration suggesting Fe(II) oxidation is coupled to a process called dissimilatory nitrate reduction to NH_4_^+^ (equation 6). In our study nitrate reduction in the autotrophic condition was only about 0.2 mM. If this was partially converted to NH_4_^+^ it would fall within the technical error of the measurement (the used medium consists of ∼5 mM NH_4_^+^) and thus lays under the detection limit (Fig. S1).


(6) \begin{eqnarray*}
4{\mathrm{Fe}}\left( {{\mathrm{II}}} \right) + {\mathrm{N}}{{\mathrm{O}}_3}^ - + 10{{\mathrm{H}}^ + } \to 4{\mathrm{Fe}}\left( {{\mathrm{III}}} \right) + {\mathrm{N}}{{\mathrm{H}}_4}^ + + 3{{\mathrm{H}}_2}{\mathrm{O}}.
\end{eqnarray*}

Given the observed Fe(II) oxidation to NO_3_⁻-reduction ratio of 10.68, some electrons derived from Fe(II) oxidation may have been diverted to additional metabolic processes other than nitrate-reducing Fe(II) oxidation. This additional Fe(II) oxidation coexisting with nitrate-reducing Fe(II) oxidation however was not observed in the Fe(II) only control. Further studies to better quantify nitrate reduction to NH_4_^+^ or N_2_O, could be achieved by labelled NO_3_^−^ (^15^NO_3_^−^) which helps to discriminate formed NH_4_^+^ from NH_4_^+^ from the medium itself.

### Nitrate-reducing Fe(II) oxidation under autotrophic conditions was not sustainable

After transferring the 9-days old autotrophic culture to fresh medium containing Fe(II) and NO_3_⁻, no further Fe(II) oxidation was observed within 9 days (second transfer) (Fig. 3A). The culture was subsequently transferred again to fresh medium, and Fe(II) oxidation was still not observed within another 9 days of incubation (third transfer) (Fig. 3A). Reduction of NO_3_⁻ was under the detection limit (Fig. 3B), however the bacteria did show formation of N_2_O up to 9.10±0.42 µM after the second transfer and 14.10±3.91 µM after the third transfer (Fig. 3C).

This N_2_O formation might be due to slight denitrification activity (nitrate reduction under the detection limit) using trace organic carbon in the medium (Rittenberg 1972, Jakus et al. 2021a) and may have been required by the bacterium for cell maintenance.

In the experiment by Weber et al. (2006b), cultures were incubated with acetate, Fe(III) and NO_3_^−^, which led to the reduction of Fe(III) to Fe(II). After acetate was depleted and Fe(III) reduction had ceased, Fe(II) oxidation was initiated by spiking cultures with NO_3_^−^. In this case, the metabolome released by the bacteria was still present at the phase of Fe(II) oxidation. In contrast, we washed the culture grown with acetate/NO_3_^−^ and transferred the cells into fresh medium containing Fe(II) and NO_3_^−^ (first transfer). Both approaches, the one used by Weber et al. (2006b) and our setup, resulted in comparable Fe(II) oxidation rates, i.e. Weber et al. study: 1.25 × 10^−8^ mmol cell^−1^ day^−1^ and our study: 2.33 × 10^−8^ mmol cell^−1^ day^−1^. However, our experiments, including successive transfers, suggest the possibility that stored organic carbon within the cells (from the previous incubation with acetate/NO_3_^−^) affects Fe(II) oxidation, as later transfers, in which the bacteria probably were depleted in this stored carbon, did not show any Fe(II) oxidation anymore.

This effect [Fe(II) oxidation in the first generation, inoculated from heterotrophic cultures, but not in later generations] has been observed in several non-autotrophic nitrate-reducing Fe(II)-oxidizers (Bryce et al. 2018), including *Thiobacillus denitrificans* (Becker et al. 2025), *Pseudogulbenkiania ferrooxidans* (Chen et al. 2018), and *Acidovorax* sp. BoFeN1 (Becker and Kappler 2026), underscoring the importance of successive transfer experiments under identical conditions to accurately assess physiological capacities.

### Cell viability of *G. metallireducens* under autotrophic, nitrate-reducing, Fe(II)-oxidizing conditions

**Figure 4:**
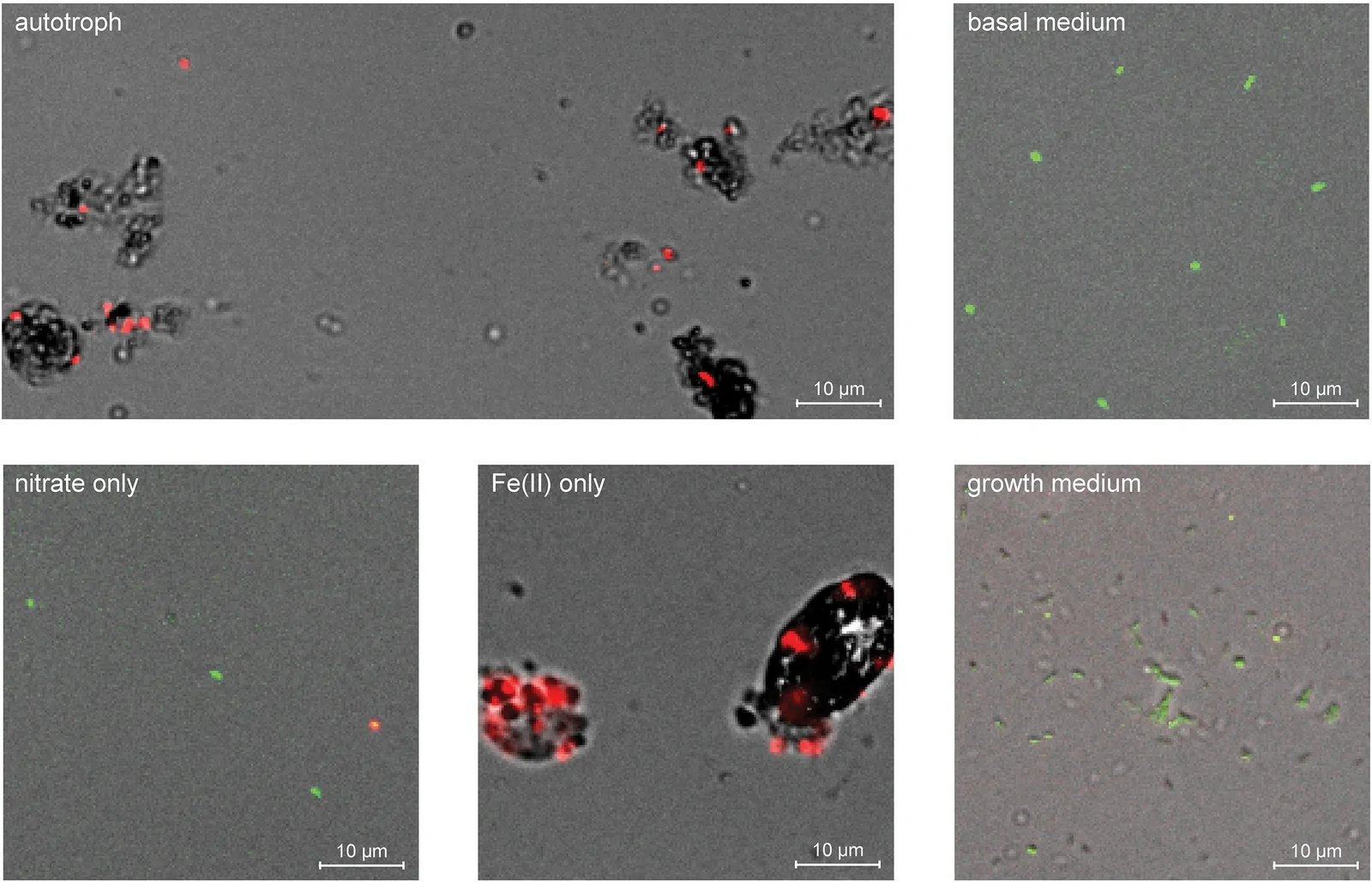
Micrographs showing cell viability by Live/Dead staining of *G. metallireducens* cultures 9 days after inoculation with 2.2 × 10^6^ (±17%) cells ml^−1^ from a heterotrophically grown culture (Fig. 1, first transfer). Red indicates defective cells and green intact cells. Autotroph: NO_3_⁻-reducing Fe(II)-oxidizing cultures under autotrophic condition (10 mM Fe(II) and 4 mM NO_3_⁻); Basal medium: without electron donor or acceptor; Fe(II) only: 10 mM Fe(II); nitrate only: 4 mM NO_3_⁻; growth medium: 10 mM acetate and 20 mM NO_3_⁻.

To evaluate cell viability after 9 days of incubation under autotrophic conditions, we used Live/Dead staining. Cells treated with Fe(II) were stained solely with propidium iodide (PI), indicating compromised cell membranes and cell death. Although Syto9 (stains intact and defective cells) stained cells were detected when incubated with basal medium (without electron donor and acceptor), with NO_3_^⁻^ only and with acetate-/nitrate-containing growth medium (Fig. 4), they were less bright compared to heterotrophically grown *G. metallireducens* harvested from the exponential phase as used for the inoculation of the experiment (Fig. S2, first transfer suspension). In brief, *G. metallireducens* did not stain well with Syto9 after incubation of 9 days and did show a Syto9 fluorescence signal when incubated with Fe(II). This could be due to phenotypic differences, for example the production of conductive pili, which may hypothetically interfere with staining by preventing dye entry into the cells and subsequent binding to DNA.

Attempts to use flowcytometry-based cell couniting of *G. metallireducens* incubated with Fe(II) were unsuccessful. We tried untreated cells and fixation methods such as 10% ethanol, 10% formalin, 2.5% polyformaldehyde, and heat treatment (10 min at 70°C) in combination with Syto9, PI, BacLight™ and SYTOX™ Green staining, using oxalic solution to dissolve iron-minerals (Becker et al. 2025).

Nevertheless, we conducted cell counting by flow cytometry of samples taken from incubations with basal medium, NO_3_^⁻^ only and in the acetate-/NO_3_^⁻^-containing growth medium of thefirst transfer where the inoculum was coming from heterotrophic conditions without Fe(II) (Fig. S2A). We did not observe any population increase in the basal medium without an electron donor or acceptor present, or in the NO_3_^⁻^ only medium without an electron donor. This was not expected, as impurities in the medium, such as dissolved organic matter, can promote slight increases in cell numbers following transfer to fresh medium [Rittenberg 1972, Jakus et al. 2021a, Becker and Kappler 2026 (submitted to FEMS)]. However, this effect does not appear to occur for *G. metallireducens*. The acetate-/NO_3_**^⁻^**-containing growth medium supported growth as expected, indicating that the bacterial culture used in our experiment was intact and not harmed by cell washing before inoculation.

As an indicator for the viability of the inoculated cells of each of the three transfers under autotrophic conditions, we quantified nitrate reduction of subsamples incubated in acetate-/NO_3_**^⁻^**-containing growth medium. We observed typical nitrate reduction in cultures inoculated with cell suspensions prepared for the first transfer and second autotrophic transfer but not for the third autotrophic transfer (Fig. S2B). This indicates that the nitrate-reducing, Fe(II)-oxidizing cell culture incubated under autotrophic conditions for 9 days and used for the second transfer was still intact. However, the culture used for the third transfer, after a total of 18 days under autotrophic conditions, became defective. This suggests that the cells indeed suffered from the incubation with Fe(II) only as sole electron donor.

### Conclusion—*G. metallireducens* is a non-autotrophic nitrate-reducing Fe(II)-oxidizer

In our experiments with *G. metallireducens*, NO₃⁻ reduction and Fe(II) oxidation occurred only when both substrates were present, confirming that *G. metallireducens* performs nitrate-reducing Fe(II) oxidation. This finding is consistent with previous studies (Weber et al. 2006b, Finneran et al. 2002). However, successive transfers under autotrophic conditions demonstrated that the Fe(II) oxidation observed in the first autotrophic generation inoculated by an acetate-/NO_3_⁻-grown preculture was not sustainable. Fe(II) oxidation could not be maintained in later autotrophic transfers, indicating that nitrate-reducing Fe(II) oxidation does not take place independently of prior organic carbon availability. After incubation for 18 days under autotrophic Fe(II)-/NO₃⁻-containing conditions, *G. metallireducens* cells showed signs of impairment, as indicated by their failure to initiate nitrate reduction upon transfer back to acetate-/NO₃⁻-containing medium (monitored for 9 days). This further evidences that *G. metallireducens* cannot sustain as autotrophic NRFeOx.

We therefore classify *G. metallireducens* as a non-autotrophic nitrate-reducing Fe(II)-oxidizer. Whether *G. metallireducens* conserves energy from nitrate-reducing Fe(II) oxidation or can use Fe(II) as a growth-supporting lithotrophic electron donor remains unresolved. Consequently, further physiological designations, such as mixotrophy, cannot be conclusively assigned at this stage. This question could be addressed through comparative experiments conducted with and without Fe(II) as an additional lithotrophic electron donor, combined with ATP measurements and quantitative PCR to assess energy conservation and population dynamics.

Our findings have important implications for fundamental research conducted under autotrophic or oligotrophic conditions in which nitrate-reducing Fe(II) oxidation is investigated, including studies related to early Earth analog environments, Mars-relevant systems, and nitrate remediation in organic-poor subsurface aquifers. For modeling nitrate removal in organic-limited environments, the use of a truly autotrophic nitrate-reducing Fe(II)-oxidizer would provide a more accurate representation of natural processes.

We therefore do not recommend the use of *G. metallireducens* under strictly organic carbon–limited (autotrophic) conditions. Instead, microbial communities that have been shown to sustain autotrophic nitrate-reducing Fe(II) oxidation, such as cultures KS (previously called Straub culture), BP, AG, and HP, may represent more suitable model systems (Tominski et al. 2018, Huang et al. 2021a, 2021b, Jakus et al. 2021a, 2021b, Grimm et al. 2024).

## Supplementary Material

fiag098_Supplemental_File

## References

[bib1] Anderson L. Simultaneous spectrophotometric determination of nitrite and nitrate by flow injection analysis. Anal Chim Acta. 1979;110:123–8. 10.1016/S0003-2670(01)83537-6.

[bib2] Balch WE, Fox GE, Magrum LJ et al. Methanogens: reevaluation of a unique biological group. Microbiol Rev. 1979;43:260–96. 10.1128/mr.43.2.260-296.1979. PMC281474 390357

[bib3] Becker S, Dang TT, Wei R et al. Evaluation of *Thiobacillus denitrificans*’ sustainability in nitrate-reducing Fe(II) oxidation and the potential significance of Fe(II) as a growth-supporting reductant. FEMS Microbiol Ecol. 2025;101:fiaf024. 10.1093/femsec/fiaf024. PMC11963766 40097297

[bib4] Becker S, Enright AML, Metals KA. Microbes, and Minerals—the Biogeochemical Side of Life. In: Kroneck P, Torres M. S. (eds.), Berlin, Germany: De Gruyter, 2021;185–228. 10.1515/9783110589771-013

[bib5] Becker S, Kappler A. *Pseudogulbenkiania ferrooxidans* strain 2002—evaluation of its sustainability in nitrate-reducing Fe(II) oxidation under autotrophic conditions. 2026. unpublished manuscript. 10.1093/femsec/fiag098 PMC13599779 42667377

[bib6] Bruce RA, Achenbach LA, Coates JD. Reduction of (per)chlorate by a novel organism isolated from paper mill waste. Environ Microbiol. 1999;1:319–29. 10.1046/j.1462-2920.1999.00042.x. 11207750

[bib7] Bryce C, Blackwell N, Schmidt C et al. Microbial anaerobic Fe(II) oxidation—ecology, mechanisms and environmental implications. Environ Microbiol. 2018;20:3462–83. 10.1111/1462-2920.14328. 30058270

[bib8] Chauhan A, Patzner MS, Bhattacharyya A et al. Interactions between iron and carbon in permafrost thaw ponds. Sci Total Environ. 2024;946:174321. 10.1016/j.scitotenv.2024.174321. 38942322

[bib9] Chen D, Liu T, Li X et al. Biological and chemical processes of microbially mediated nitrate-reducing Fe(II) oxidation by *Pseudogulbenkiania* sp. strain 2002. Chem Geol. 2018;476:59–69. 10.1016/j.chemgeo.2017.11.004.

[bib10] Childers SE, Ciufo S, Lovley DR. *Geobacter metallireducens* accesses insoluble Fe(III) oxide by chemotaxis. Nature. 2002;416:767–9. 10.1038/416767a. 11961561

[bib11] Drabesch S, Lechtenfeld OJ, Bibaj E et al. Climate induced microbiome alterations increase cadmium bioavailability in agricultural soils with pH below 7. Commun Earth Environ. 2024;5:637. 10.1038/s43247-024-01794-w.

[bib12] Finneran KT, Housewright ME, Lovley DR. Multiple influences of nitrate on uranium solubility during bioremediation of uranium-contaminated subsurface sediments. Environ Microbiol. 2002;4:510–6. 10.1046/j.1462-2920.2002.00317.x. 12220407

[bib13] Forster P, Storelvmo T, Armour K et al., the Earth’s Energy Budget, Climate Feedbacks, and Climate Sensitivity. In: Climate Change 2021: the Physical Science Basis. Contribution of Working Group I to the Sixth Assessment Report of the Intergovernmental Panel on Climate Change. Cambridge University Press (Cambridge, UK). 2021, 923–1054. 10.1017/9781009157896.009.

[bib14] Granger J, Sigman DM. Removal of nitrite with sulfamic acid for nitrate N and O isotope analysis with the denitrifier method. Rapid Commun Mass Spectrom. 2009;23:3753–62. 10.1002/rcm.4307. 19908214

[bib15] Grimm H, Lorenz J, Straub D et al. Nitrous oxide is the main product during nitrate reduction by a novel lithoautotrophic iron(II)-oxidizing culture from an organic-rich paddy soil. Appl Environ Microbiol. 2024;91:e01262–24. 10.1128/aem.01262-24. PMC11784278 39641603

[bib16] Huang YM, Jakus N, Kleindienst S et al.‘*Candidatus* ferrigenium straubiae’ sp. nov., ‘*Candidatus* ferrigenium bremense’ sp. nov., ‘*Candidatus* ferrigenium altingense’ sp. nov., are autotrophic Fe(II)-oxidizing bacteria of the family *Gallionellaceae*. Syst Appl Microbiol. 2021a;45:126306. 10.1016/j.syapm.2022.126306. 35279466

[bib17] Huang YM, Straub D, Kappler A et al. A novel enrichment culture highlights core features of microbial networks contributing to autotrophic Fe(II) oxidation coupled to nitrate reduction. Microb Physiol. 2021b;31:280–95. 10.1159/000517083. 34218232

[bib18] Jakus N, Blackwell N, Osenbrück K et al. Nitrate removal by a novel lithoautotrophic nitrate-reducing, iron(II)-oxidizing culture enriched from a pyrite-rich limestone aquifer. Appl Environ Microbiol. 2021a;87:e00460–21. 10.1128/AEM.00460-21. PMC8373248 34085863

[bib19] Jakus N, Mellage A, Höschen C et al. Anaerobic neutrophilic pyrite oxidation by a chemolithoautotrophic nitrate-reducing iron(II)-oxidizing culture enriched from a fractured aquifer. Environ Sci Technol. 2021b;55:9876–84. 10.1021/acs.est.1c02049. 34247483

[bib20] Jamieson J, Prommer H, Kaksonen AH et al. Identifying and quantifying the intermediate processes during nitrate-dependent iron(II) oxidation. Environ Sci Technol. 2018;52:5771–81. 10.1021/acs.est.8b01122. PMC6427828 29676145

[bib21] Jokai K, Nakamura T, Okabe S et al. Simultaneous removal of nitrate and heavy metals in a continuous flow nitrate-dependent ferrous iron oxidation (NDFO) bioreactor. Chemosphere. 2021;262:127838. 10.1016/j.chemosphere.2020.127838. 32768756

[bib22] Kappler A, Bryce C, Mansor M et al. An evolving view on biogeochemical cycling of iron. Nat Rev Micro. 2021;19:360–74. 10.1038/s41579-020-00502-7. 33526911

[bib23] Klueglein N, Kappler A. Abiotic oxidation of Fe(II) by reactive nitrogen species in cultures of the nitrate-reducing Fe(II) oxidizer *Acidovorax* sp. BoFeN1–questioning the existence of enzymatic Fe(II) oxidation. Geobiology. 2013;11:180–90. 10.1111/gbi.12019. 23205609

[bib24] Lovley DR, Giovannoni SJ, White DC et al. *Geobacter metallireducens* gen. nov. sp. nov., a microorganism capable of coupling the complete oxidation of organic compounds to the reduction of iron and other metals. Arch Microbiol. 1993;159:336–44. 10.1007/BF00290916. 8387263

[bib25] Lovley Derek R, Stolz JF, Nord GL et al. Anaerobic production of magnetite by a dissimilatory iron-reducing microorganism. Nature. 1987;330:252–4. 10.1038/330252a0.

[bib26] Qi M, Li Y, Fu Y et al. Enhanced coupling of Fe(II) oxidation and nitrate reduction benefits chemoautotrophic carbon fixation in estuarine and coastal sediments. Environ Sci Technol. 2025;59:21189–201. 10.1021/acs.est.5c05446. 40994108

[bib27] Ravishankara AR, Daniel JS, Portmann RW. Nitrous oxide (N_2_O): the dominant ozone-depleting substance emitted in the 21st century. Science (New York, N.Y.). 2009;326:123–5. 10.1126/science.1176985. 19713491

[bib28] Rittenberg SC. The obligate autotroph—the demise of a concept. Antonie Van Leeuwenhoek. 1972;38:457–78. 10.1007/BF02328114.

[bib29] Rivett MO, Buss SR, Morgan P et al. Nitrate attenuation in groundwater: a review of biogeochemical controlling processes. Water Res. 2008;42:4215–32. 10.1016/j.watres.2008.07.020. 18721996

[bib30] Schaedler F, Kappler A, Schmidt C. A revised iron extraction protocol for environmental samples rich in nitrite and carbonate. Geomicrobiol J. 2018;35:23–30. 10.1080/01490451.2017.1303554.

[bib31] Senko JM, Stolz JF. Evidence for iron-dependent nitrate respiration in the dissimilatory iron-reducing bacterium *Geobacter metallireducens*. Appl Environ Microb. 2001;67:3750–2. 10.1128/AEM.67.8.3750-3752.2001. PMC93084 11472960

[bib32] Skeggs LT. An automatic method for colorimetric analysis. Am J Clin Pathol. 1957;28:311–22. 10.1093/ajcp/28.3_ts.311. 13458160

[bib33] Sørensen J, Thorling L. Stimulation by lepidocrocite (7-FeOOH) of Fe(II)-dependent nitrite reduction. Geochim Cosmochim Acta. 1991;55:1289–94. 10.1016/0016-7037(91)90307-Q.

[bib34] Stookey LL. Ferrozine-a new spectrophotometric reagent for iron. Anal Chem. 1970;42:779–81. 10.1021/ac60289a016.

[bib35] Tai YL, Dempsey BA. Nitrite reduction with hydrous ferric oxide and Fe(II): stoichiometry, rate, and mechanism. Water Res. 2009;43:546–52. 10.1016/j.watres.2008.10.055. 19081595

[bib36] Tominski C, Heyer H, Lösekann-Behrens T et al. Growth and Population Dynamics of the Anaerobic Fe(II)-Oxidizing and Nitrate-Reducing Enrichment Culture KS. Appl Environ Microbiol. 2018;84:e02173–17. 10.1128/AEM.02173-17. PMC5930324 29500257

[bib37] Weber KA, Pollock J, Cole KA et al. Anaerobic nitrate-dependent iron(II) bio-oxidation by a novel lithoautotrophic betaproteobacterium. Strain 2002. 2006a;72:686–94. 10.1128/AEM.72.1.686-694.2006. PMC1352251 16391108

[bib38] Weber KA, Urrutia MM, Churchill PF et al. Anaerobic redox cycling of iron by freshwater sediment microorganisms. Environ Microbiol. 2006b;8:100–13. 10.1111/j.1462-2920.2005.00873.x. 16343326

[bib39] Widdel F, Bak F. Gram-negative mesophilic sulfate-reducing bacteria. In: Balows A, Trüper HG, Dworkin M et al. (eds.), The Prokaryotes: a Handbook on the Biology of Bacteria: ecophysiology, Isolation, Identification, Applications. New York, NY: Springer, 1992, 3352–78. 10.1007/978-1-4757-2191-1_21

[bib40] Zhang M, Li Y, Long X et al. An alternative approach for nitrate and arsenic removal from wastewater via a nitrate-dependent ferrous oxidation process. J Environ Manage. 2018;220:246–52. 10.1016/j.jenvman.2018.05.031. 29783178

[bib41] Zhang X, Li A, Szewzyk U et al. Improvement of biological nitrogen removal with nitrate-dependent Fe(II) oxidation bacterium *Aquabacterium parvum* B6 in an up-flow bioreactor for wastewater treatment. Bioresour Technol. 2016;219:624–31. 10.1016/j.biortech.2016.08.041. 27544912

