# Supplementary material for: Geobacter metallireducens—evaluation of its sustainability in nitrate-reducing Fe(II) oxidation under autotrophic conditions
Source: FEMS Microbiol Ecol. 2026 Aug 29;102(10):fiag098. doi: 10.1093/femsec/fiag098 (PMC13599779; doi:10.1093/femsec/fiag098)
Supplement: Supplementary file 1 [file fiag098_supplemental_file.pdf]

SUPPLEMENTAL INFORMATION

Geobacter metallireducens—evaluation of its sustainability in nitrate-reducing Fe(II) oxidation under autotrophic conditions

Stefanie Becker[1], Lukas A. Ostler[1], Andreas Kappler[1†]*

[1]Geomicrobiology, Department of Geosciences, University of Tübingen, Schnarrenbergstrasse 94–96, D-72076 Tübingen, Germany

[†]Cluster of Excellence: EXC 3121: TERRA – Terrestrial Geo-Biosphere Interactions in a Changing World, University of Tübingen, Germany

[*]To whom correspondence should be sent:

Stefanie Becker, E-Mail: stefanie.becker@geo.uni-tuebingen.de

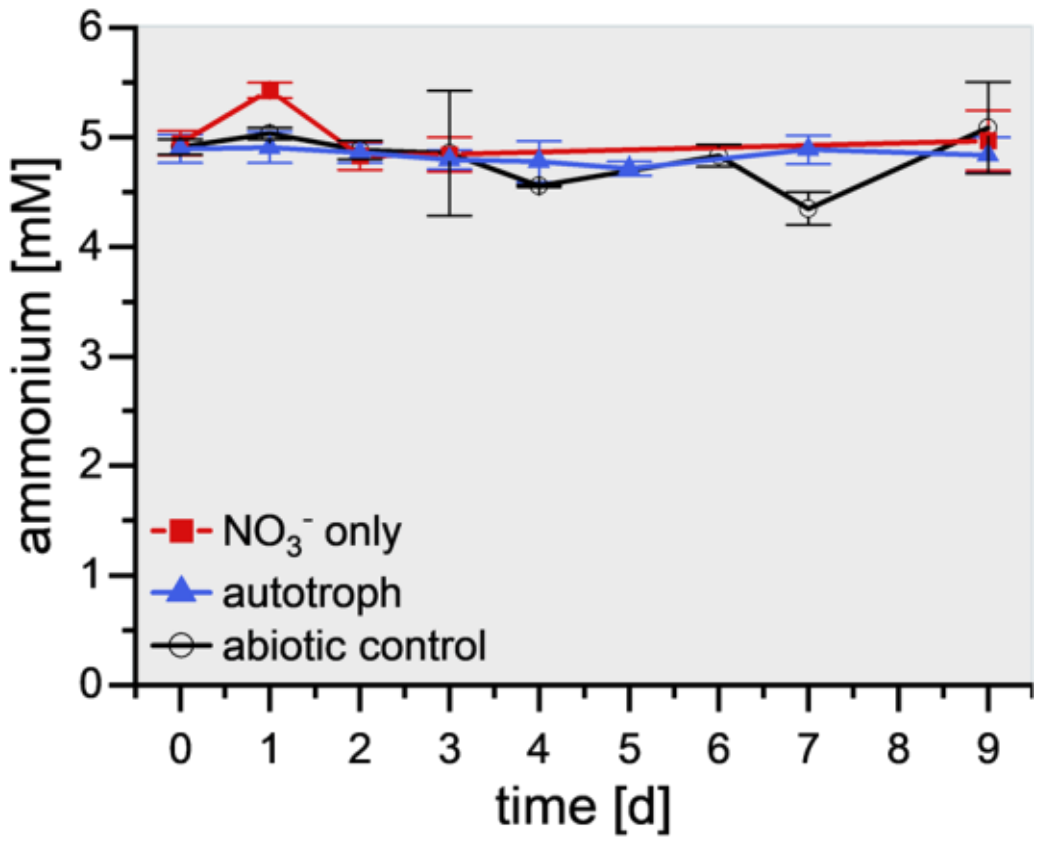

Figure S1:Ammonium concentration in G. metallireducens cultures incubated autotrophically in medium containing 10 mM Fe(II) and 4 mM NO_3^- and in medium containing only NO_3^- (no electron donor). Not inoculated medium containing 10 mM Fe(II) and 4 mM NO_3^- functioned as abiotic control. The cultures were inoculated with washed cells (~2.2×10^6 cells mL^{-1}) of a heterotrophically grown preculture (medium containing 10 mM acetate, 20 mM NO_3^-). Error bars indicate ±1 standard deviation calculated from triplicate measurements.

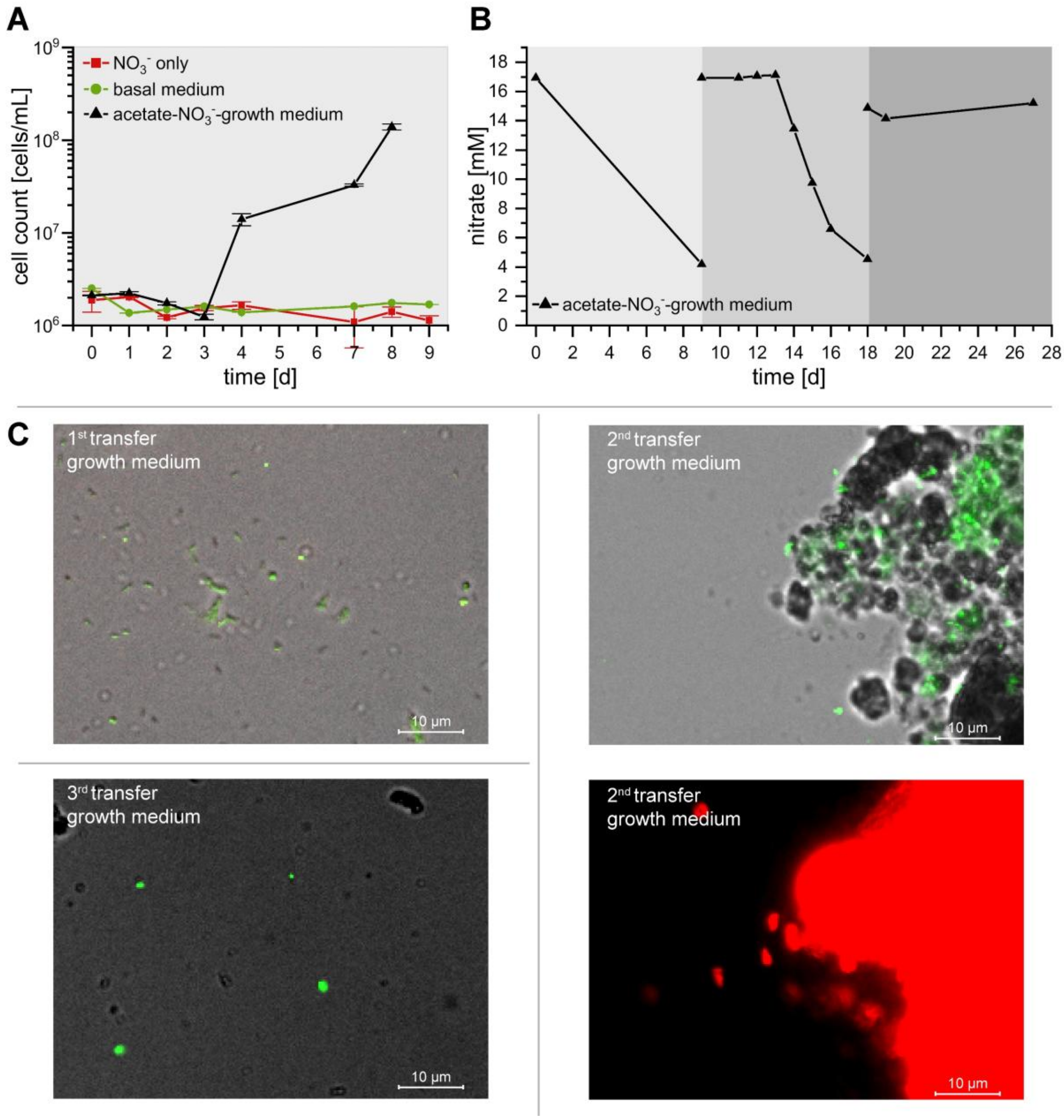

Figure S2: Viability controls of evaluating G. metallireducens sustainability in nitrate-reducing, Fe(II)-oxidizing autotrophic conditions. (A) Cell growth of cultures incubated in medium containing no electron donor or acceptor (basal medium), medium containing only NO_3^- (no electron donor) and growth supporting medium containing 10 mM acetate and 20 mM NO_3^-. Error bars indicate ±1 standard deviation calculated from triplicate measurements. (B) Nitrate reduction as indication for cell activity. Light gray background: 1st transfer from a heterotrophically grown preculture (medium containing 10 mM acetate and 20 mM NO_3^-). Middle gray background: 2nd transfer from a nitrate-reducing, Fe(II) oxidizing culture incubated on autotrophic conditions [medium containing 10 mM Fe(II) and 4 mM NO_3^-] for 9 days. Dark gray background: 3rd transfer from a nitrate-reducing, Fe(II) oxidizing culture incubated on autotrophic conditions for 18 days. (C) Micrographs showing Live/Dead staining of G. metallireducens in growth supporting medium, 9 days after the respective transfer. Red indicates defective cells and green intact cells. In contrast to the other images, the second transfer is shown without an overlay; instead, the propidium iodide and SYTO9 channels are displayed separately, as the fluorescence signal from the minerals would otherwise overlap with the SYTO9 signal.

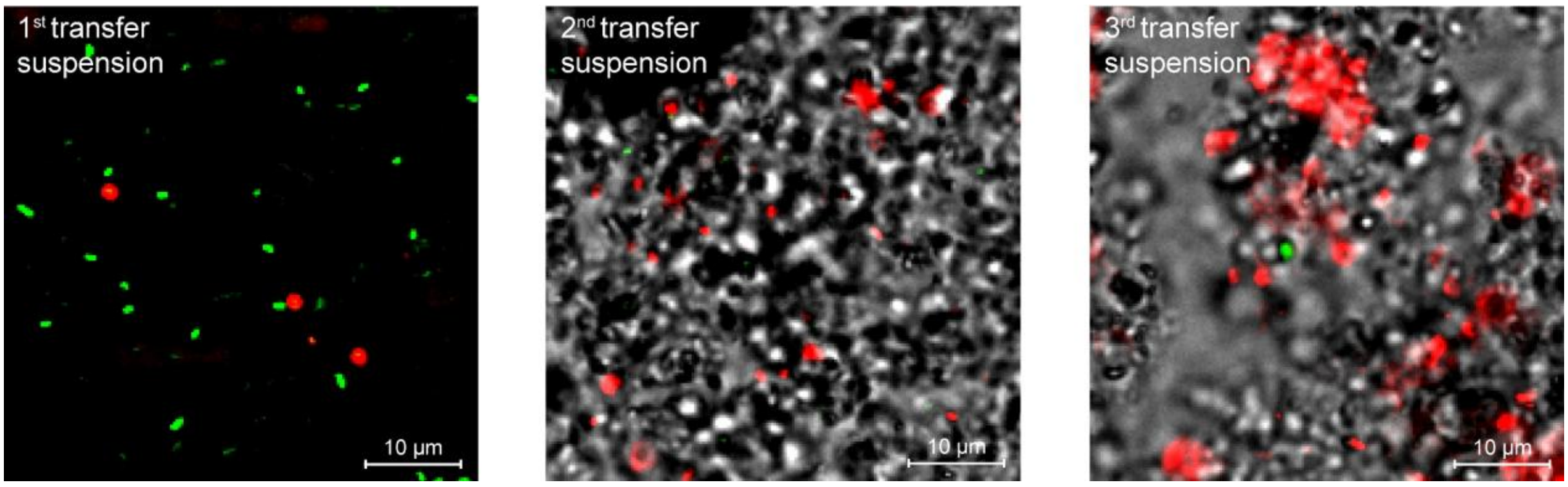

Figure S3: Micrographs showing Live/Dead staining of G. metallireducens as used to inoculate the three phases of the experiment (Figure 1, main text). Washed cells were suspended in basal medium without substrates (no electron donor or acceptor). Cell suspension for the 1st transfer stemming from a heterotrophically grown preculture (medium containing 10 mM acetate and 20 mM NO_3^-) harvested at the exponential phase. Cell suspension for the 2nd transfer steamed from a nitrate-reducing, Fe(II) oxidizing culture incubated on autotrophic conditions [medium containing 10 mM Fe(II) and 4 mM NO_3^-] for 9 days. Cell suspension for the 3rd transfer stemming from a 18-day old culture on autotrophic conditions.
